# 851. Neuroinflammation After Burn Injury

**DOI:** 10.1093/jbcr/irag033.321

**Published:** 2026-04-06

**Authors:** Alexandra Halevi, Andrew Hoisington, Thomas O Vogler, Christopher Stamper, Lisa Brenner, Kevin M Najarro, Rachel H McMahan, Cameron Gibson, Juan-Pablo Idrovo, Arek J Wiktor, Fred Endorf, Elizabeth J Kovacs

**Affiliations:** University of Colorado Anschutz, Aurora, Colorado; University of Colorado Anschutz, Aurora, Colorado; University of Colorado Anschutz, Aurora, Colorado; University of Colorado Anschutz, Aurora, Colorado; University of Colorado Anschutz, Aurora, Colorado; University of Colorado Anschutz, Aurora, Colorado; University of Colorado Anschutz, Aurora, Colorado; UCHealth Burn and Frostbite Center - Anschutz Medical Campus, Aurora, Colorado; University of Colorado Anschutz, Aurora, Colorado; UCHealth Burn and Frostbite Center - Anschutz Medical Campus, Aurora, Colorado; UCHealth Burn and Frostbite Center - Anschutz Medical Campus, Aurora, Colorado; University of Colorado Anschutz, Aurora, Colorado

## Abstract

**Introduction:**

Burn trauma induces a complex inflammatory response, including poorly-understood neuroinflammation. Our understanding of the effect of burn injury on neuroinflammation in patients is severely limited. The purpose of this study is to look at plasma markers of neuroinflammation in patients with acute burn injuries.

**Methods:**

Participants were recruited from 2 large urban academic hospitals, both American Burn Association accredited burn centers. Blood samples were collected from patients seen in the admitted to the burn unit within 24 hours of acute thermal burn. Plasma markers include ubiquitin C-terminal hydrolase L1 (UCH-L1), glial fibrillary acidic protein (GFAP), and neurofilament light (NfL). These were compared to each patient’s revised Baux Score as a surrogate marker for severity of injury. A random Forest Model was used to assess predictability of dependent outcomes including length of stay (LOS), days in the intensive care unit (ICU), and days on the ventilator. Linear mixed effect model methodology controlling for sex and ethanol use was used to determine the relationship between revised Baux Score (total burn surface area [%] + age[years] + 17[if inhalation injury]) and neuroinflammatory markers. Non-parametric data are presented as median [Q1-Q3].

**Results:**

A total of 63 patients were enrolled in the study with a median TBSA of 20% [10-40], and median age of 47 years old [36-54]. 81% of enrolled patients were male, and the median revised Baux score was 76 [58-93]. The patients had a median LOS of 24 days [15-42], 10 days in the ICU [7-25], and 1 day on the ventilator [0-10]. 41% of the patients were ethanol positive on plasma testing. When comparing neuroinflammatory markers and Revised Baux Score, all three were significantly related: UCH-L1 (*p*<.001), GFAP (*p*=.008), and NfL (*p*=.002).

**Conclusions:**

This study demonstrates early increases in neuroinflammatory markers that correlate with revised Baux Score, which is a well-validated risk model for mortality in burn patients. These findings show a relationship between revised Baux score (a composite of age and burn size) and central nervous system inflammation. Additionally, the data suggest that neuroinflammation may represent a critical and underrecognized component of the burn-injury pathway, and may act as a target for future therapeutic interventions aimed at improving recovery after burn injury.

**Applicability of Research to Practice:**

These findings demonstrate neuroinflammation is a systemic consequence of burn injury and linked to predictors of mortality. This underscores the potential utility of neuroinflammatory markers in improving our understanding of burn injury and as a potential value for prognostication and potential treatment.

**Funding for the study:**

NIH.

